# Lower protection of cytological screening for adenocarcinomas and shorter protection for younger women: the results of a case–control study in Florence

**DOI:** 10.1038/sj.bjc.6601754

**Published:** 2004-03-30

**Authors:** M Zappa, C B Visioli, S Ciatto, A Iossa, E Paci, P Sasieni

**Affiliations:** 1 Clinical and Descriptive Epidemiological Unit, CSPO (Centro per lo Studio e la Prevenzione Oncologica), Via di San Salvi, 12, Florence 50135, Italy; 2 Cancer Research UK Department of Epidemiology, Mathematics and Statistics, Wolfson Institute of Preventative Medicine, London, UK

**Keywords:** case–control, cervical screening, cervical cancer, cervical adenocarcinoma

## Abstract

The efficacy of cytological screening in preventing adenocarcinoma of cervix uteri as compared to squamous cell cancer has been evaluated by means of a case–control study in the province of Florence. The odds ratios of women who had a Pap test within the 3 years before the index date was 0.65 (95% confidence interval (CI) 0.26–1.64) and 0.15 (95% CI 0.07–0.31), for adenocarcinoma and squamous cancer, respectively. The duration of the protective effect was shorter in women below the age of 40 years than in older women.

Although the efficacy of cytological screening for cervical cancer is beyond doubt (mortality and incidence have been falling dramatically in Western Europe since screening has been adopted on a large scale), the optimal resolution of several aspects of cervical screening (e.g. compliance, criteria for referral, cost-effectiveness) is still awaited. Recently, two problematic aspects concerning Pap smear efficacy have been highlighted:

1. Incidence rates of cervical adenocarcinomas are increasing despite extensive screening (the reverse is true for squamous carcinoma) (Smith *et al*, 2000), and cytological screening seems to be less effective for adenocarcinoma than for squamous carcinoma.
2. Screening efficacy seems to be age dependent, and a shorter rescreening interval has been suggested for younger women (Sasieni *et al*, 2003).

The present case–control study aimed to evaluate the efficacy of cytological screening in preventing adenocarcinoma of the cervix as compared to squamous carcinoma, and in preventing cervical cancer in younger (<40 years) as compared to older (⩾40 years) women.

## MATERIALS AND METHODS

The Centro per lo Studio e la Prevenzione Oncologica (CSPO) has been running organised population-based cervical screening in the Florence District since 1980. Screening details have been described previously (Palli *et al*, 1990). A computerised archive collects Pap smears that are read as well as the results obtained in screen positive subjects referred and attending diagnostic assessment at CSPO. Using several data sources, we estimated that about two-third of all Pap smears (in a public or in a private setting) performed in the screening area are archived in our centre. Information on high-grade cervical intraepithelial neoplasias and cervical cancers incident in screened subjects is obtained from a population-based registry (Tuscany Tumour Registry: RTT), incidence data available up to December 1999.

### Cases

All women aged below 70 years registered at RTT as having a cervical cancer diagnosed between 1994 and 1999 were considered. Microinvasive carcinomas were excluded as well as nine cases in women who had been resident in the screening area for less than 5 years. All records were reviewed to assess histological type (squamous, adenocarcinoma, other, not specified). The date of diagnosis (as reported in the RTT) was used as the index date of the matched set in the case–control study.

### Controls

Four controls for each case were randomly selected from the municipality residence database among women matched by age (same year of birth), who were alive at the index date of the matched case. Women were eligible as controls if they had been resident in the screening area for at least 5 years and if no hysterectomy was known before the index date. Information on hysterectomy was obtained from a database available in the screening programme, which includes women reporting hysterectomy when referring for Pap test and, more recently, those recorded in local hospital records provided by the Tuscany Region. This archive may be incomplete, but lack of information on hysterectomy would conservatively affect the evaluation of protection by the Pap test (hysterectomised women cannot receive a Pap test).

### Screening history

All Pap tests archived in the screening database were considered (positive or negative). In order to exclude smears taken because of symptoms, smears taken within 12 months before the index date of matched cases were ignored (for both cases and controls). Four categories were defined for screening history analysis: (a) at least one Pap test within 3 years before the index date; (b) most recent Pap test more than 3 years, but less than 6 years before the index date; (c) most recent Pap test more than 6 years before the index data; and (d) no Pap test recorded in the database.

### Statistical analysis

Using conditional logistic analysis in the STATA software, we estimated the odds ratios (OR) of developing invasive cancer associated with screening history. Separate analysis was undertaken for squamous cancer and adenocarcinomas, as well as for younger (<40 years) and older woman (⩾40 years). Covariates introduced in the models were place of birth (Italy/outside) and civil status (ever married *vs* never married), both variables being available in the municipality residence database.

## RESULTS

A total of 208 cases and 832 matched controls were selected. Among cases, 148 (71.1%) had squamous carcinoma, 53 (25.5%) adenocarcinoma and seven (3.4%) other or unspecified types. Ever married women were slightly more frequent among controls than among cases (88.6 *vs* 86.1%, *P*=0.315), whereas women born outside Italy were less frequent among controls as compared to cases (0.7 *vs* 3.4%, *P*=0.002).

Table 1

**Table 1:** Distribution by age of cases (overall and according to histological type) and controls. Adjusted (for civil status and place of birth) OR of developing a fully invasive squamous cancer (relative to controls) associated with having been screened in the previous 5 years

**Age group**		**Histological type (%)**				
**(years)**	**Controls (%)**	**Squamous (%)**	**Adenocarcinomas (%)**	**NOS and others (%)**	**All cases (%)**	**OR^b^ for squamous cancers (95%CI)**
<40	207 (24.9)	30 (20.3)	20 (37.7)	2 (28.6)	52 (25.0)	0.32 (0.11–0.95)
40–49	250 (30.0)	50 (33.8)	11 (20.7)	1 (14.3)	62 (29.8)	0.11 (0.04–0.33)
50–59	163 (19.6)	28 (18.9)	11 (20.7)	2 (28.6)	41 (19.7)	0.08 (0.02–0.31)
60–69	212 (25.5)	40 (27.0)	11 (20.7)	2 (28.6)	53 (25.5)	0.22 (0.06–0.83)
All ages	832 (100)	148 (71.1)	53 (25.5)	7 (3.4)	208 (100)	0.17 (0.10–0.30)

a Excluding tests performed the year before the index date.

b *Baseline*: never screened (OR=1.0). CI=confidence interval; OR=odds ratio; NOS=not otherwise specified.

shows the distribution by age group of cases (overall and according to histological type) and controls. Adenocarcinomas occurred more frequently in younger women as compared to squamous cancers (*P*=0.045). In the same table are reported OR (and 95% Confidence Interval – 95% CI) for squamous carcinoma (relative to controls) associated with having been screened in the previous 5 years. A marked increase is evident in the level of protection for women older than 40 years.

Table 2

**Table 2:** Distribution of the cases (overall including not otherwise specified (NOS) and other cancers and divided by squamous cancer and adenocarcinomas) and controls by history of screening. OR of developing a fully invasive cervical cancer by time since last test, adjusted for civil status and birth place

	**Controls**	**Squamous**		**Adenocarcinoma**		**All cases**	
**Time since last test^a^**	**No. (%)**	**No. (%)**	**OR (95% CI)**	**No. (%)**	**OR (95% CI)**	**No. (%)**	**OR (95% CI)**
<3 years	187 (22.5)	11 (7.4)	0.15 (0.07–0.30)	9 (17.0)	0.65 (0.26–1.65)	22 (10.6)	0.25 (0.15–0.42)
3–<6 years	171 (20.5)	11 (7.4)	0.20 (0.10–0.39)	13 (24.5)	0.99 (0.43–2.29)	26 (12.5)	0.34 (0.21–0.56)
⩾6 years	237 (28.5)	48 (32.4)	0.56 (0.36–0.87)	10 (18.9)	0.54 (0.24–1.23)	59 (28.4)	0.56 (0.38–0.82)
Never screened	237 (28.5)	78 (52.7)	1.0^b^	21 (39.6)	1.0^b^	101 (48.6)	1.0^b^
OR=odds ratios; CI=confidence interval.							

a Excluding tests performed the year before the index date.

b Baseline.

shows the screening history of cases (according to histological type) and controls and the risk of developing a fully invasive cervical cancer (squamous or adenocarcinoma) as a function of time since last test. Overall, no Pap smear was recorded in 52.7% of squamous, 39.6% of adenocarcinomas and 28.5% of controls (*P*<0.001). Odds ratios were adjusted for civil status and place of birth. Women who had a Pap test within 3 years before the index date had an OR=0.24 (95% CI=0.14–0.41) as compared to never screened subjects. The corresponding value for squamous cancer was 0.15 (95% CI=0.07–0.31) and that for adenocarcinomas was much higher (OR=0.65; 95% CI=0.26–1.64), but not statistically significant.

Table 3

**Table 3:** OR of developing a fully invasive cervical cancer (overall including not otherwise specified (NOS) and other cancers and divided by squamous cancer and adenocarcinomas) by time since last test and by age group (<40, >=40 years), adjusted for civil status and place of birth

**Time since last test^a^**	**Squamous (95% CI)**	**Adenocarcinoma (95% CI)**	**All cases (95% CI)**
*<40 years*			
1–<3 years	0.16 (0.03–0.77)	0.76 (0.18–3.11)	0.35 (0.13–0.95)
3–<6 years	0.45 (0.14–1.48)	1.24 (0.26–5.86)	0.63 (0.26–1.52)
⩾6 years	0.68 (0.23–2.01)	1.03 (0.25–4.25)	0.83 (0.37–1.86)
1–<5 years	0.32 (0.11–0.95)	0.82 (0.24–2.79)	0.51 (0.23–1.11)
⩾5 years	0.51 (0.19–1.41)	1.25 (0.32–4.90)	0.71 (0.33–1.54)
Never screened	1.0	1.0	1.0
			
*⩾40 years*			
1–<3 years	0.15 (0.07–0.33)	0.56 (0.16–2.01)	0.22 (0.12–0.42)
3–<6 years	0.14 (0.06–0.33)	0.78 (0.27–2.28)	0.26 (0.14–0.48)
⩾6 years	0.53 (0.32–0.88)	0.42 (0.14–1.24)	0.48 (0.31–0.74)
1–<5 years	0.14 (0.07–0.27)	0.67 (0.23–1.93)	0.24 (0.14–0.40)
⩾5 years	0.47 (0.29–0.77)	0.49 (0.18–1.30)	0.44 (0.29–0.68)
Never screened	1.0	1.0	1.0

a Excluding tests performed the year before the index date. OR=odds ratios; CI=confidence interval.

presents the results of multivariate analysis of the risk of developing a fully invasive cervical cancer (squamous or adenocarcinoma) as a function of time since the last test and of age for two different strata of age (<40 *vs* ⩾40 years). We considered either a 3-year interval or a 5-year interval in order to have more stable ORs. The ORs for invasive squamous cancer was similar within 3 years before the index date (0.16 95% CI=0.03–0.77 or 0.15 95% CI=0.07–0.33) for women <40 or ⩾40 years, respectively), whereas it was higher for younger women having the last Pap test >3 years and <6 years before the index date (0.45 95% CI 0.14–1.48 for women aged <40 or 0.14 95% CI=0.06–0.33 for women aged ⩾40, respectively).

## DISCUSSION

Although case–control studies have been extensively used for evaluating screening for cervical cancer, this approach has been criticised on the grounds that a bias in favour of screening could occur (Sasieni, 2001, pp 93–105; Zappa and Ciatto, 2001, pp 99–118). In the present study, nevertheless, the main aim was not to compare the protection in screened as compared to unscreened woman: on the contrary, we compared the relative protective effect of screening (a) for different histological type of cancer (squamous and adenocarcinomas) or (b) in different age groups, and we suppose that any potential bias should similarly affect different compared strata. In other words, we cannot exclude that our case–control approach may overestimate the effect of screening, but we do not expect such an overestimate to be different when comparing adenocarcinomas to squamous cancers or younger to older women. However, other differential biases are possible. If adenocarcinoma is more likely to be screen detected, this would bias results in favour of a preventive effect on adenocarcinoma (cases will have a full screening interval, whereas controls will on average be halfway between two screens); if high-risk young women are more likely to attend whereas high-risk older women are more likely to refuse screening, then such a difference could explain the result we have observed.

The present study shows a lower protection from cytological screening for adenocarcinoma than squamous carcinoma. The result is consistent across age groups, and confirms the findings of other case–control studies (Mitchell *et al*, 1995; Sato *et al*, 1997). From a public health point of view, this might be of minor concern as adenocarcinomas currently account for less than 25% of cervical cancers, but such a lower protective effect of screening should be kept in mind, considering that the incidence of adenocarcinoma is increasing in younger women. The investigation of the possible causes of a lower protective effect of cytological screening from adenocarcinoma (e.g. lower sensitivity of cytology, different sensitivity of smear sampling techniques, differences in sojourn time intrinsic of adenocarcinoma) is essential to support further action.

As far as the association of age with screening efficacy is concerned, we found that the relative protection of screening from invasive squamous carcinoma is shorter in younger (<40 years) than in older (⩾40 years) women. This finding confirms the results of a recent large study carried out in UK (Sasieni *et al*, 2003), which recommended a reduction from 5 to 3 years in the rescreening interval for women below 50 years of age. In Italy, a 3-year rescreening interval is recommended at any age, and such an interval seems to grant sufficient protection. In the case of older age groups, our findings suggest that a longer interval (5 years) could be safely adopted.

The scenario of cervical cancer prevention is open to marked changes, due to the widely recognised aetiological role of human papilloma virus (HPV) and due to the possibility of the future use of HPV vaccination as a radical alternative to screening; but the role of HPV testing as a possible screening tool should not be dismissed. Attempts to improve screening sensitivity and efficacy in specific circumstances (younger age, adenocarcinoma) should not be based only on a more aggressive cytological approach (e.g. reduced rescreening interval, lower threshold for cytological abnormality prompting diagnostic assessment) but also consider the opportunity for adding HPV testing to Pap smear as a screening tool.
